# First Genome Sequence of a Mexican Multidrug-Resistant *Acinetobacter baumannii* Isolate

**DOI:** 10.1128/genomeA.00156-16

**Published:** 2016-03-24

**Authors:** Lucía Graña-Miraglia, Luis Lozano, Semiramis Castro-Jaimes, Miguel A. Cevallos, Patricia Volkow, Santiago Castillo-Ramírez

**Affiliations:** a Programa de Genómica Evolutiva, Centro de Ciencias Génomicas, Universidad Nacional Autónoma de México, Cuernavaca, Morelos, México; b Instituto Nacional de Cancerología, Ciudad de México, D.F., México

## Abstract

*Acinetobacter baumannii* has emerged as an important nosocomial pathogen worldwide. Here, we present the draft genome of the first multidrug-resistant *A. baumannii* isolate, sampled from a tertiary hospital in Mexico City. This genome will provide a starting point for studying the genomic diversity of this species in Mexico.

## GENOME ANNOUNCEMENT

*Acinetobacter baumannii*, a Gram-negative coccobacillus, has surfaced as one of the leading causes of hospital-acquired infections in recent years (1, 2). Remarkably, many strains of this species have multidrug-resistant phenotypes showing resistance against most antibiotic classes (2). Ever since the publication of the first genome sequence of an *A. baumannii* isolate a considerable amount of genome sequences have been published. As of 23 November 2015, there are slightly over 30 complete genomes and more than a thousand draft genomes publically available. However, none of them has come from Mexico. Here, in order to start exploring the genome diversity of this species in Mexico, we report the first genome sequence of the Mexican isolate *A. baumannii* Mex11594, hereinafter referred to as Mex11594.

Mex11594 was isolated on 17 June 2011 at the Instituto Nacional de Cancerología (Mexico’s National Institute of Oncology), a tertiary hospital located in Mexico City, from pleural fluid of a male patient. Multilocus sequence typing (the Oxford scheme) on this strain revealed that it belongs to the sequence type (ST) 231 (3). According to the PubMLST database (4), isolates sharing this ST have been found in Argentina, Brazil, France, Germany, the Netherlands, and United States, implying that this ST is not exclusive to Latin America. The Mex11594 genome was sequenced using an Illumina MiSeq platform with a 300-bp library (250-bp paired-end reads). The sequencing run produced 1,631,060 reads that were assembled into a preliminary assembly using Velvet version 1.2.10 (5) and SPAdes (6). In order to improve our assembly, we used Consed version 23.0 (7) for gap closure, assembly editing, and error correction. The improved assembly yielded 12 contigs and had a total size of 3,745,499 bp, with an *N*_50_ of 684,685 bp, an average coverage of 41× and a G+C content of 39.03%. Given the number of contigs, the *N*_50_, and the average coverage, we are confident that this is a good assembly that should be helpful for future comparative genome analysis. Our improved assembly suggested the existence of several plasmids, which was corroborated via an Eckhardt gel analysis (8). The chromosome was composed of 8 contigs and had an estimated size of 3,700,249 bp.

The draft genome was annotated by means of the Rapid Annotations Using Subsystems Technology (RAST) (9) server, which predicted 3,488 coding sequences, 772 of them hypothetical proteins. Ten rRNAs and 51 tRNAs were identified. This is a multidrug-resistant isolate (10), as it has molecular determinants conferring resistance to at least 3 different types of antibiotics. For instance, this isolate is nonsusceptible to meropenem and imipenem (carbapenems), tobramycin (aminoglycosides), levofloxacin (fluoroquinolones), and ceftazidime and ceftriaxone (cephalosporins) to name but a few.

 Further and more extensive comparative analysis of this and other Mexican isolates will help to unveil the genomic diversity (as well as the genetic components conferring the multidrug-resistant phenotypes) of this bacterial pathogen in Mexico and Latin America and, hence, to better understand its worldwide diversity.

### Nucleotide sequence accession number. 

The *A. baumannii* MEX11594 genome sequence was deposited in GenBank under the accession number LQXZ00000000**.**

## References

[1] Antunes LC, Visca P, Towner KJ 2014 *Acinetobacter baumannii*: evolution of a global pathogen. Pathog Dis 71:292–301. doi:10.1111/2049-632X.12125. 24376225

[2] Zarrilli R, Pournaras S, Giannouli M, Tsakris A 2013 Global evolution of multidrug-resistant *Acinetobacter baumannii* clonal lineages. Int J Antimicrob Agents 41:11–19. doi:10.1016/j.ijantimicag.2012.09.008. 23127486

[3] Bartual SG, Seifert H, Hippler C, Luzon MA, Wisplinghoff H, Rodríguez-Valera F 2005 Development of a multilocus sequence typing scheme for characterization of clinical isolates of *Acinetobacter baumannii*. J Clin Microbiol 43:4382–4390. doi:10.1128/JCM.43.9.4382-4390.2005. 16145081 PMC1234098

[4] Jolley KA, Maiden MC 2010 BIGSdb: scalable analysis of bacterial genome variation at the population level. BMC Bioinformatics 11:595. doi:10.1186/1471-2105-11-595. 21143983 PMC3004885

[5] Zerbino DR, Birney E 2008 Velvet: algorithms for de novo short read assembly using de bruijn graphs. Genome Res 18:821–829. doi:10.1101/gr.074492.107. 18349386 PMC2336801

[6] Bankevich A, Nurk S, Antipov D, Gurevich AA, Dvorkin M, Kulikov AS, Lesin VM, Nikolenko SI, Pham S, Prjibelski AD, Pyshkin AV, Sirotkin AV, Vyahhi N, Tesler G, Alekseyev MA, Pevzner PA 2012 SPAdes: a new genome assembly algorithm and its applications to single-cell sequencing. J Comput Biol 19:455–477. doi:10.1089/cmb.2012.0021. 22506599 PMC3342519

[7] Gordon D, Green P 2013 *Consed*: a graphical editor for next-generation sequencing. Bioinformatics 29:2936–2937. doi:10.1093/bioinformatics/btt515. 23995391 PMC3810858

[8] Eckhardt T 1978 A rapid method for the identification of plasmid desoxyribonucleic acid in bacteria. Plasmid 1:584–588. doi:10.1016/0147-619X(78)90016-1. 107540

[9] Overbeek R, Olson R, Pusch GD, Olsen GJ, Davis JJ, Disz T, Edwards RA, Gerdes S, Parrello B, Shukla M, Vonstein V, Wattam AR, Xia F, Stevens R 2014 The SEED and the Rapid Annotation of microbial genomes using Subsystems Technology (RAST). Nucleic Acids Res 42:D206–D214. doi:10.1093/nar/gkt1226. 24293654 PMC3965101

[10] Magiorakos AP, Srinivasan A, Carey RB, Carmeli Y, Falagas ME, Giske CG, Harbarth S, Hindler JF, Kahlmeter G, Olsson-Liljequist B, Paterson DL, Rice LB, Stelling J, Struelens MJ, Vatopoulos A, Weber JT, Monnet DL 2012 Multidrug-resistant, extensively drug-resistant and pandrug-resistant bacteria: an international expert proposal for interim standard definitions for acquired resistance. Clin Microbiol Infect 18:268–281. doi:10.1111/j.1469-0691.2011.03570.x. 21793988

